# AMBA CUP: Ensuring Accuracy in Measurement of Volume of Water for Salt Sugar Solution or Oral Rehydration Solution Preparation in Diarrhea Management

**DOI:** 10.5334/aogh.4301

**Published:** 2023-10-05

**Authors:** Aniekan Jumbo Etokidem

**Affiliations:** 1 Department of Community Medicine, University of Calabar/University of Calabar Teaching Hospital, Calabar, Cross River State, NG

**Keywords:** diarrhea, oral rehydration therapy, oral rehydration solution, salt sugar solution, volume of water, Amba Cup

## Abstract

Diarrhea remains a major cause of under-five mortality globally. In 2016, it accounted for 8% of under-five mortality worldwide. Most of these deaths occur in developing countries. Fluid replacement using Oral Rehydration Solution (ORS) or Salt Sugar Solution (SSS), has been the mainstay of diarrhea management. Gaps in knowledge and practice regarding the preparation of these solutions have been identified by various researchers. One challenge encountered by healthcare providers and caregivers of under-five children has been lack of a standard, easy to clean cup for measurement of accurate volume of water for ORS or SSS preparation. Soft drink bottles, which are currently being used, are difficult to clean because of their narrow necks. More so, the size and volume of these bottles change so often that non-numerate caregivers get easily confused. The aim of this paper is to introduce the AMBA CUP, an easy-to-clean cup that can be used to accurately measure one litre of water for SSS or ORS preparation.

## Background

In 2016, diarrhea accounted for about 8% of under-five mortality, translating to 1,300 under-five deaths daily or 480,000 children yearly worldwide. Most of these deaths occurred in South-East Asia and sub-Saharan Africa [1]. In Nigeria, diarrhea accounted for 10% of under-five mortality in 2016 [1].

Fluid replacement using Oral Rehydration Solution (ORS) or Salt Sugar Solution (SSS), has been the mainstay of diarrhea management and has saved the lives of millions of children worldwide [2].

In Nigeria, when healthcare providers give caregivers health education regarding preparation of Salt Sugar Solution or Oral Rehydration Solution, they tell them to measure I liter of water using two big soft drink bottles. Recently, the rate at which soft drink producers change the sizes of their bottles has brought confusion to both healthcare providers and caregivers. The situation is worse with caregivers who have not received formal education and may, therefore, not be able to read the volume labels on these changing bottle sizes. Currently, the commonest big soft drink bottle in Nigeria has a volume of 600ml. Measuring water with two of this (or twice with one) would give 1.2 liters. Preparing the SSS or ORS with this would lead to a hypo-osmolar solution. Another problem with the soft drink bottle is that it is difficult to wash when it is dirty. This is because it is not possible for one’s hand to pass through the narrow neck of the bottle so as to scrub the inside.

## Gaps Identified by Previous Research

Several researchers had documented gaps in knowledge and practice of caregivers regarding the preparation of ORS or SSS. Some of these gaps pertain to measurement of the correct volume of water. They therefore documented the need for a standard one-litre container for measuring water for ORS or SSS preparation [3,4,5].

## The AMBA Cup

The AMBA CUP^1^ is a calibrated cup that can be used to measure exactly one liter (1 L) of water. In order to assist non-numerate caregivers, the 1 Liter point is indicated with a red mark. It is very easy to wash compared to soft drink bottles. Because the AMBA CUP can be used for some other domestic purposes like drinking water, tea and for measuring water for cooking, it will not just be abandoned and forgotten, like the soft drink bottle, only to be frantically searched for when a child has diarrhea. Most households dispose of soft drink bottles immediately after drinking the contents. Sometimes, a used soft drink bottle may not be found and, if found, may be too dirty to be used immediately. Even after washing, the cleanliness of the bottle cannot be guaranteed.

The AMBA CUP is a handy solution to Salt Sugar Solution or Oral Rehydration Solution preparation.

## Footnotes

Adapted, with permission, from an existing 1.5L cup made by DANA PLAST Limited, Lagos, Nigeria. The cup will be made available for distribution at health centers in Calabar, Nigeria, at the initial stage with scale up to other parts of the country later. Presently, the product from which the AMBA CUP is adapted costs 250 Nigerian Naira (0.60 US Dollars). Users and reviewers have suggested that the cup should have a cover. This will be considered in the next generation of AMBA Cup.
